# Evaluating the capacity of the distress thermometer to detect high fear of cancer recurrence

**DOI:** 10.1002/pon.6066

**Published:** 2022-11-24

**Authors:** Esther Deuning‐Smit, José A. E. Custers, Linda Kwakkenbos, Rosella P. M. G. Hermens, Judith B. Prins

**Affiliations:** ^1^ Department of Medical Psychology Radboud Institute for Health Sciences Radboud University Medical Center Nijmegen The Netherlands; ^2^ Department of IQ Healthcare Radboud Institute for Health Sciences Radboud University Medical Center Nijmegen The Netherlands; ^3^ Clinical Psychology Radboud University Nijmegen The Netherlands; ^4^ Department of Psychiatry Radboudumc Center for Mindfulness Radboud Institute for Health Sciences Radboud University Medical Center Nijmegen The Netherlands

**Keywords:** cancer, detection, distress thermometer, fear of cancer recurrence, oncology, questionnaire, screening, self‐report measure, survivorship

## Abstract

**Objectives:**

Fear of cancer recurrence (FCR) is common and burdensome to patients, but often remains undetected. Oncology professionals report need for tools to improve FCR detection in routine care. Oncology care guidelines recommend the Distress Thermometer (DT) for distress screening, but it has not been validated for FCR. This study evaluated the capacity of the DT and accompanying problem list to detect FCR.

**Methods:**

Amalgamated data of two studies with 149 breast cancer and 74 colorectal cancer survivors were used. We evaluated the Dutch DT including the DT score, problem list *fears* item and emotional domain score using Receiver Operating Characteristic analyses. The Dutch Cancer Worry Scale‐6 (CWS‐6) was used as reference measure, with validated cut‐off scores ≥10 and ≥12 for high FCR. Sensitivity, specificity, negative and positive predictive values were calculated.

**Results:**

The DT score showed poor performance in discriminating between low and high FCR. The recommended cut‐off ≥4 had low sensitivity (65% for CWS‐6≥10; 72% for CWS‐6 ≥12) and specificity (67% and 58%). No other cut‐off had an acceptable combination of sensitivity and specificity. The fears item had low sensitivity (29% and 44.9%) and high specificity (95% and 94%). The emotional domain score had fair performance in discriminating between low and high FCR but there was no cut‐off with acceptable sensitivity and specificity.

**Conclusion:**

The DT as currently recommended in oncology care guidelines is not suitable to effectively detect FCR in routine care. To improve patients access to psychosocial care, it should be investigated how FCR‐specific measures can be integrated in oncology practice.

## 1 INTRODUCTION

Fear of cancer recurrence (FCR),^1^ defined as ‘fear, worry, or concern relating to the possibility that cancer will come back or progress’^2^ is an important unmet need for people affected by cancer.^3^ FCR can be conceptualized on a continuum from low transient levels enabling healthy lifestyle adaptations and adequate symptom monitoring, to clinical FCR^2^ that is associated with a low quality of life^4^, ^5^, ^6^ and increased healthcare utilisation.^7^ The psychosocial care needs vary along this continuum, as those with lower FCR levels could benefit from psycho‐education and minimal interventions while those with high or clinical levels might need treatment by psycho‐oncology professionals.^8^ This advocates the need for stepped or matched care models that tailor the intensity and nature of psychosocial support to patients' FCR severity.^9^, ^10^ These models can only be successful if FCR is adequately detected by physicians and nurses in oncological follow‐up care.^9^ However, implementation of FCR interventions is currently hampered by low referral rates^11^, ^12^ and therefore improving FCR detection is one of the priorities of the international FCR research agenda.^13^ There are several possible explanations for low referral rates. Patients may not report FCR due to unawareness of FCR interventions and reluctance to discuss psychological problems with their physician or nurse.^11^, ^14^, ^15^ Barriers for physicians and nurses include the lack of knowledge and skills and low self‐efficacy related to FCR detection and referral. As FCR is unrelated to disease and treatment characteristics in most studies^6^ identification of those at risk of high FCR is complicated. Clinicians express a need for tools to differentiate between mild adaptive FCR and more severe levels of FCR.^11^, ^15^, ^16^ Routine screening with a validated measure may help to adequately identify patients with high FCR and allocate the limited psychological treatment resources to those patients who may benefit most.

Multiple measures have been developed to assess FCR severity.^4^, ^17^ While FCR‐specific questionnaires are valuable for research and clinical use within psycho‐oncology services (e.g., routine outcome monitoring), the feasibility to use them as a screening instrument in daily oncology follow‐up care is questioned.^15^ Cancer survivors face many physical and psychosocial challenges and physicians and nurses have limited time in medical consultations for psychosocial screening.^11^ Therefore, it is useful to explore whether general psychosocial screening instruments that are already commonly used in cancer follow‐up are suitable to detect high FCR.^9^

The *distress thermometer* (DT) is a single item self‐report measure introduced by the National Comprehensive Cancer Network (NCCN) practice guideline^18^ and adopted in clinical guidelines in other countries, including the Netherlands.^19^ Patients rate their overall distress on a visual analogue scale and indicate the source of distress on the accompanying problem list, with items in 5 life domains. A cut‐off score of ≥4 is most often recommended in studies^20^, ^21^ and guidelines,^18^, ^19^ but other cut‐offs have been suggested,^20^ dependent on language, cancer population, reference measure and time point.^21^ Clinical guidelines recommend that a ‘positive screen’ is followed by a conversation about the distress severity and marked items on the problem list, and, if necessary, additional problem‐specific assessment and referral to a mental health or other supportive care professional depending on the reported problems. There are several knowledge gaps pertaining the suitability of the DT to detect FCR. First, it has not yet been investigated whether the DT score is able to differentiate between low and high FCR. Second, on the problem list an item indicative for high FCR is missing. The emotional domain includes a *fears* item, but this item may be too general to detect FCR.^15^ Finally, a validation study suggested that the problem list domains could function as a scale, when adding the number of problems selected.^1^ The *emotional domain score* may be suitable to detect FCR, but this has not been investigated.

This study aims to evaluate the capacity of the DT score, the fears item and the emotional domain score to detect high FCR. As recommendations regarding the optimal DT cut‐off score varies across studies, we will examine cut‐off score ≥4 as well as other potential cut‐off scores. A better understanding of the capacity of the DT to detect high FCR will guide implementation of psychological interventions for FCR in daily clinical practice.

## 2 METHODS

### 2.1 Patients and procedure

This study used data from two studies in breast cancer survivors (BCS) and colorectal cancer survivors (CRCS). The first study (*n* = 150) was a multicenter randomized controlled parallel‐group trial evaluating the efficacy of the BREATH eHealth self‐management intervention compared to usual care.^22^, ^23^ Participants were women with a histologically proven malignancy of the breast and 2–4 months after completing curative‐intent primary treatment. Data were collected between August 2010 and March 2012. Participants provided written informed consent and ethical approval was obtained by the coordinating center (Radboud University Medical Center Medical Review Ethics Committee No. 2009/144) and all participating centers (Netherlands Trial Register NTR2935). The response rate in this study was 89%. A detailed description of the study has been published.^22^, ^23^ The second study (*n* = 80) was a cross‐sectional study assessing the prevalence and characteristics of FCR in long‐term CRCS.^24^ CRCS were treated with curative intent, disease‐free (no recurrence or metastases), and 1–9 years after completing surgery. Data were collected in March 2012. Participants provided written informed consent and ethical approval was obtained by the coordinating center (Radboud University Medical Center Medical Review Ethics Committee No. 2011/562). The response rate in this study was 36%. Non‐responders were significantly older than responders (*t* (209) = 2.0, *p* = 0.046) and there was no difference in gender. A detailed description of the study has been published.^24^ The current study used DT and CWS‐6 scores from both studies. For the BCS sample, baseline data, collected before random assignment, were analyzed.

### 2.2 Measures

The DT includes a single‐item DT score and a problem list. The DT score is a visual‐analogue scale (0 = no distress; 10 = extreme distress) shaped like a thermometer indicating the level of distress experienced in the past week. The problem list indicates the source of distress with dichotomous items (yes/no) in 5 domains: practical, social, emotional, spiritual and physical. We used the validated Dutch DT^1^ which has an adapted problem list (47 items) compared to the original version (46 items). The emotional domain contains 10 items (depression, fears, nervousness, *emotional control*, *intrusions, self‐esteem*, *loneliness*, concentration, *guilt* and *loss of control*; italic items were adapted in the Dutch version), had good reliability in the current study sample using the KR20 formula (0.81) and correlated strongly with the DT score (*r* = 0.57). A DT score cut‐off of ≥5 was recommended in the original Dutch validation study, but cut‐of scores of ≥3,^25^ ≥4^26^ and ≥7^27^ have been suggested. The most recent Dutch practice guideline recommends a cut‐off of ≥4, in accordance with the NCCN guidelines.^18^

*The 6‐item Cancer Worry Scale (CWS‐6)* was the reference measure for FCR. Items are rated on a 4‐point Likert scale. Total scores range from 6 to 24, with higher scores indicating higher FCR. The Dutch version of the CWS‐6 has been validated in a Dutch sample of 1033 cancer patients and survivors with gastro‐intestinal stromal tumors, colorectal, breast, and prostate cancer.^28^ Two cut‐offs have been validated: a score ≥10 suitable for screening purposes (sensitivity 82%, specificity 83%) and a score ≥12 to detect FCR levels that may indicate need for referral to a high‐intensity treatment (sensitivity 88%, specificity 73%). The reliability in this sample was good (Cronbach's α = 0.89).

### 2.3 Statistical analyses

Participants with a missing CWS‐6 total score or with missing DT score, fears item and emotional domain score were excluded from the analyses. The emotional domain score was calculated by summing the number of items with a positive response. Mean scores were calculated for the CWS‐6, DT score and emotional domain score and a frequency for the ‘fears’ item. Descriptive statistics were used to characterize participants' socio‐demographic and clinical characteristics. Differences between the two subsamples on the outcome variables were explored using *t*‐tests and chi‐square tests. The associations of the CWS‐6 with the DT score and emotional domain score were analyzed with bivariate correlations. Receiver operating characteristic (ROC) curves and their 95% confidence interval (CI) were used to assess the suitability of the DT score and the emotional domain score to detect high FCR based on the CWS‐6 (score ≥10 or score ≥12). The area under the ROC curve (AUC) indicates overall performance (excellent ≥0.90; good 0.80–0.89; fair 0.70–0.79; poor ≤0.69). Sensitivity, specificity, negative predictive value (NPV) and positive predictive value (PPV) were calculated for all potential DT and emotional domain score cut‐offs and for the fears item. Sensitivity and specificity indicated the adequacy of the DT relative to the CWS‐6, where sensitivity is the proportion of people with high FCR and specificity the proportion of people with low FCR that are correctly identified by the DT. The usefulness of the DT in clinical practice is largely determined by PPV, the probability that people with a positive DT result have high FCR, and NPV, the probability that people with a negative DT result have low FCR. Which values are acceptable for these indices is highly context‐specific.^29^ In the context of oncology follow up care the sensitivity and NPV of a screening instrument for FCR should be maximized to avoid many patients with high FCR being missed while maintaining sufficient specificity and PPV as follow‐up assessment of those with a positive screen requires scarce resources (e.g., time and costs). In the current study we therefore strived for a sensitivity, NPV, specificity and PPV around 0.80. Additional analyzes were performed to determine the capacity for a combination of the thermometer score and the fears item.

In a sensitivity analysis, it was explored whether the results of the total sample were reflected in the two samples separately.

## 3 RESULTS

Among 230 participants, five were excluded from the analyses because of missing CWS‐6 total score and two were excluded with missing DT score, emotional domain score and fears item. In total, 223 participants were included in the analyses: 149 BCS and 74 CRCS. Demographic and disease characteristics and mean CWS‐6 and DT scores are displayed in Table 1. The mean age was 56.3 years and 82% was female. The mean DT score was 3.92 out of 10, 19.5% selected ‘yes’ on the fears item and the mean emotional domain score was 2.92 out of 10. The mean CWS‐6 total score was 10.66 out of 24. On the CWS‐6, 61% scored above the cut‐off ≥10 and 35% above the cut‐off ≥12.

**TABLE 1:** Demographic, medical and psychological characteristics

	Colorectal *n* = 74	Breast *n* = 149	Total *n* = 223		
	Mean (SD)	Mean (SD)	Mean (SD)		
Age, years	67.32 (11.93)	50.83 (8.76)	56.30 (12.59)		
Time since diagnosis, years	4.87 (2.31)	n.a.	n.a.		

	N (%)	N (%)	N (%)		
Female	34 (45.9)	149 (100)	183 (82.1)		
Nationality					
Dutch	73 (100)	148 (99.3)	221 (99.5)		
other	0 (0)	1 (0.7)	1 (0.5)		
Education					
Primary	19 (26.0)	27 (18.1)	46 (20.7)		
Secondary	28 (38.4)	79 (53.0)	107 (48.2)		
Tertiary	26 (35.6)	43 (28.9)	69 (31.1)		
Disease stage					
I	13 (18.6)	n.a.	n.a.		
II	28 (40.0)	n.a.	n.a.		
III	29 (41.4)	n.a.	n.a.		
IV	0 (0.0)	n.a.	n.a.		
Treatment					
Surgery only	48 (64.9)	0 (0)	48 (21,5)		
Surgery and chemotherapy	22 (29.7)	144 (96.6)	166 (74.4)		
Surgery and radiotherapy	8 (10.8)	109 (73.2)	117 (52.5)		
Hormone treatment	n.a.	98 (65.8)	n.a.		
Herceptin	n.a.	30 (20.1)	n.a.		
Prior psychological treatment	21 (30.0)	n.a.	n.a.		
Randomization (CBT)	n.a.	70 (47.0)	n.a.		

	Mean (SD)	Mean (SD)	Mean (SD)	*t*(*p*)	Chi(*p*)
DT					
DT score	3.28 (2.63)	4.23 (2.53)	3.92 (2.60)	2.58 (0.010)	
Emotional domain	1.97 (2.22)	3.38 (2.76)	2.92 (2.67)	4.08 (<0.001)	
Fears item, N (%)	7 (9.9)	36 (24.2)	43 (19.5)		6.26 (0.012)
CWS‐6					
Total	9.08 (3.16)	11.45 (3.42)	10.66 (3.51)	4.99 (<0.001)	
Cut‐off ≥ 10, N (%)	30 (40.5)	105 (70.5)	135 (60.5)		18.54 (<0.001)
Cut‐off ≥ 12, N (%)	18 (24.3)	60 (40.3)	78 (35.0)		5.53 (0.019)

The two samples were significantly different regarding the three DT parameters and the CWS‐6. BCS had higher DT scores than CRCS (4.23 vs. 3.28), greater frequency of the fears item (24% vs. 10%), higher emotional domain scores (3.38 vs. 1.97), higher mean total CWS‐6 scores (11.45 vs. 9.08) and more often scored above the CWS‐6 cut‐off ≥10 (71% vs. 41%) and ≥12 (40% vs. 24%).

### 3.1 Performance of the DT score

Moderate correlations between the DT score and the CWS‐6 were found (Table 2). The AUC of the ROC analyses with CWS‐6 ≥10 and ≥12 were respectively 0.71 (95% CI 0.63–0.78, *p* < 0.001) and 0.69 (95% CI 0.62–0.76, *p* < 0.001), suggesting a 71% or 69% probability that a randomly selected patient defined as a ‘case’ by the CWS‐6 would score higher on the DT than a randomly selected patient defined as a non‐case. Based on the accuracy measures (sensitivity, specificity, NPV and PPV) there was no acceptable screening cut‐off for the DT score when using the CWS‐6 ≥10 and CWS‐6 ≥12 cut‐off. The DT score cut‐off ≥4 in clinical guidelines had sensitivity of 65%, specificity of 67%, PPV of 75% and NPV of 56% for CWS‐6 ≥10. For CWS‐6 ≥12, the DT ≥ 4 cutoff had 72% sensitivity, 58% specificity, 47% PPV and 80% NPV (Table 3). Lowering the DT cut‐off score to ≥3 resulted in acceptable sensitivity (77% for CWS‐6 ≥10 and 82% for CWS‐6 ≥12) but low specificity (53% CWS‐6 ≥10 and 44% CWS‐6 ≥12). Raising the score to ≥5 or higher would result in even lower sensitivity and NPV.

**TABLE 2:** Correlations between the Distress Thermometer (DT) score and emotional domain score with the Cancer Worry Scale (CWS‐6) total score

		DT		Emotional domain	
		Pearson's Correlation	*p*	Pearson's Correlation	*p*
CWS‐6	Total	*r* = 0.39 (*n* = 221)	*p* < 0.001	*r* = 0.55	*p* < 0.001
	CRCS	*r* = 0.36 (*n* = 72)	*p* < 0.001	*r* = 0.57	*p* < 0.001
	BCS	*r* = 0.36 (*n* = 149)	*p* < 0.002	*r* = 0.55	*p* < 0.001

**TABLE 3:** Accuracy measures for Distress Thermometer (DT) scores with the Cancer Worry Scale (CWS‐6) as reference standard

DT cut‐off	% Positive	CWS‐6 ≥10				CWS‐6 ≥12			
		Sens	Spec	PPV	NPV	Sens	Spec	PPV	NPV
≥1	90.0	0.970	0.205	0.648	0.818	0.961	0.131	0.367	0.864
≥2	78.3	0.887	0.375	0.682	0.688	0.921	0.290	0.405	0.875
≥3	64.7	0.767	0.534	0.713	0.603	0.816	0.441	0.434	0.821
≥4	52.5	0.654	0.670	0.750	0.562	0.724	0.579	0.474	0.800
≥5	43.0	0.549	0.750	0.768	0.524	0.605	0.662	0.484	0.762
≥6	30.3	0.391	0.830	0.776	0.474	0.434	0.766	0.493	0.721
≥7	18.6	0.226	0.875	0.732	0.428	0.303	0.876	0.561	0.706
≥8	9.5	0.128	0.955	0.810	0.420	0.184	0.952	0.667	0.690
≥9	3.6	0.053	0.989	0.875	0.408	0.079	0.986	0.750	0.671
≥10	1.4	0.023	1.000	1.000	0.404	0.026	0.993	0.667	0.661

### 3.2 Performance of the fears item

Using the CWS‐6 ≥10 cut‐off, the sensitivity of the fears item was 29.3%, the specificity was 95.4%, the PPV was 90.7% and the NPV was 46.9%. These results suggest that the fears item correctly identifies 29.3% patients with CWS‐6 ≥10 as having high FCR (sensitivity), and 95.4% patients with CWS‐6 <10 as having low fear (specificity). When patients select the fears item there is 90.7% chance that they score CWS‐6 ≥10 (PPV) and when patients do not select the fears item there is 46.9% chance they score CWS‐6 <10 (NPV). With the CWS‐6 ≥12 cut‐off, sensitivity was 44.9%, specificity 94.4%, PPV 81.4% and NPV 75.7%. A combination of any DT cutoff score with a positive response on the fears item, yielded very high specificity and PPV but very low sensitivity and NPV (Supplementary Table S1).

### 3.3 Performance of the emotional domain score

High significant correlations between the emotional domain score and the CWS‐6 were found (Table 2). The AUC of the ROC analyses with CWS‐6 ≥10 and ≥12 were respectively 0.77 (95% CI 0.71–0.83, *p* < 0.001) and 0.78 (95% CI 0.72–0.85, *p* < 0.001). Based on the accuracy measures, no acceptable cut‐off for the emotional domain score was found for CWS‐6 ≥10 and CWS‐6 ≥12. For CWS‐6 ≥10 an emotional domain cut‐off score ≥2 items had acceptable sensitivity (77%) and PVV (77%) but suboptimal specificity (64%) and NPV (64%). Lowering the cut‐off score decreased the specificity and PPV while raising the score would result in suboptimal sensitivity and NPV (Table 4). For CWS‐6 ≥12 an emotional domain cut‐off ≥3 items had acceptable sensitivity (77%) and NPV (83%) but suboptimal specificity (63%) and PPV (53%). Lowering the score further decreased the specificity and PPV while raising the score would result in suboptimal sensitivity (Table 4).

**TABLE 4:** Accuracy measures for emotional domain scores with the Cancer Worry Scale (CWS‐6) as reference standard

Emotional domain	% Positive	CWS‐6 ≥10				CWS‐6 ≥12			
		Sens	Spec	PPV	NPV	Sens	Spec	PPV	NPV
≥1	76.5	0.888	0.425	0.704	0.712	0.923	0.322	0.426	0.885
≥2	60.6	0.769	0.644	0.769	0.644	0.846	0.524	0.493	0.862
≥3	51.1	0.687	0.759	0.814	0.611	0.769	0.629	0.531	0.833
≥4	35.3	0.485	0.851	0.833	0.517	0.641	0.804	0.641	0.804
≥5	25.3	0.381	0.943	0.911	0.497	0.513	0.888	0.714	0.770
≥6	17.2	0.254	0.954	0.895	0.454	0.359	0.930	0.737	0.727
≥7	11.8	0.179	0.977	0.923	0.436	0.269	0.965	0.808	0.708
≥8	8.1	0.127	0.989	0.944	0.424	0.205	0.986	0.889	0.695
≥9	4.5	0.067	0.989	0.900	0.408	0.115	0.993	0.900	0.673
≥10	1.8	0.030	1.000	1.000	0.401	0.051	1.000	1.000	0.659

### 3.4 Explorative analyses

The performance of the DT score, fears item and emotional domain score were explored for the CRCS and BCS samples separately. For the DT score, the ROC analyses in the CRCS sample (CWS‐6 ≥10 AUC = 0.73 and CWS‐6 ≥12 AUC = 0.69) and in the BCS sample (AUC scores 0.67 and 0.67) yielded comparable results as the total sample. For both samples, no acceptable DT cut‐off to screen for FCR was found (Supplementary Tables S2 and S3). The performance of the fears item in both samples yielded similar conclusions as the results of the total sample. For the emotional domain, the ROC analyses in the CRCS sample yielded relatively better AUC scores (CWS‐6 ≥10 AUC = 0.82 and CWS‐6 ≥12 AUC = 0.82) than the performance in the BCS sample (AUC scores 0.72 and 0.75). For the CRCS sample, an emotional domain cut‐off score of ≥2 had acceptable values (sensitivity 76%, specificity 79%, PPV 71%, and NPV 83%) for CWS‐6 ≥ 10, but there was no acceptable cut‐off for CWS‐6 ≥ 12. For the BCS sample, there was no acceptable emotional domain cut‐off score (values displayed in Supplementary Tables S4 and S5).

## 4 DISCUSSION

The present study investigated the capacity of the DT and problem list to detect people with high levels of FCR, with the CWS‐6 as reference measure. Results showed that neither the DT score nor the problem list emotional domain score had a cut‐off score with acceptable sensitivity, specificity, PPV and NPV. The fears item in the problem list showed high specificity but very low sensitivity to detect high FCR.

The DT was designed to capture the broad construct of distress, while FCR is a specific psychological problem. Therefore, it is not surprising that we found low specificity and PPV to screen for FCR with the DT cut‐off score ≥4 recommended in clinical guidelines. In clinical practice, low PPV implicates that a ‘positive screen’ on the DT could indicate high FCR but can also reflect other causes of distress. More problematic was the low sensitivity of this cut‐off score, indicating that only a relatively small proportion of the patients with high FCR were detected by the DT. Possibly, FCR might not come to mind easily when individuals are asked to rate their distress on multiple life domains. These results differ from previous validation studies that found good sensitivity of the DT using the Hospital Anxiety and Depression Scale anxiety subscale (HADS‐A),^20^ however this captures a more generic anxiety construct than FCR. Using a lower DT score cut‐off ≥3 yielded acceptable sensitivity in our sample but reduced the specificity and PPV even further, while a cut‐off of ≥5 or higher resulted in low sensitivity and NPV. In terms of feasibility, lowering the cut‐off score would not be preferable as it would implicate a larger number of patients needing further assessment, of whom many will not have high FCR needing intervention.

One could argue that a low specificity of the DT score is not necessarily problematic as the problem list can identify flag areas for subsequent monitoring.^30^ Our results indicated that the problem list fears item is indeed highly specific for FCR, but has very low sensitivity and NPV. In clinical situations, this implicates that when the fears item is selected there is very high probability the person has high FCR (true positives), but also when it is not selected there is a high probability of high FCR (false negatives). The low sensitivity could result from the generic wording of the fears item. Not all individuals might recognize FCR as fear, as FCR is a multi‐componential construct which can present as (a combination of) emotions, cognitions,^2^, ^31^ images,^32^ and bodily sensations.^33^ Furthermore, the dichotomous format might make patients feel reluctant to select the fears item, as they cannot report the degree of FCR. Finally, the plural wording might make patients think they should have very high or multiple fears to select the item. The emotional domain as a scale was also not capable to detect FCR.

### 4.1 Clinical implications

Taken together it can be concluded that the DT and problem list when used according to the current clinical guidelines are not appropriate to detect FCR in clinical practice. There are several alternative possibilities to improve FCR detection. The *Screening for Distress and Referral Need* process describes an alternative use of the DT as a facilitator for communication about distress with every individual, regardless of the cut‐off score.^34^ However, FCR will likely remain undetected as this conversation is guided by the problem list which does not have an item for FCR. Another solution would be to adapt the problem list. The NCCN recently published an adapted problem list (Version 1.2022 NCCN) in which the *fears* item is changed to *fear*, and they included a *worry or anxiety* item. These items might be more sensitive to FCR, but this has yet to be investigated. Other researchers proposed to incorporate a single FCR item in the problem list, by changing *fears* to *fear of recurrence*.^15^ The Patient Concerns Inventory^35^ which is available for head and neck cancer and breast cancer follow‐up is an example of a tool that incorporates a FCR single item, and research has shown that this item is often ranked in the top 5 concerns.^36^, ^37^ However, the dichotomous format of the items in the problem list might still be inadequate to differentiate between low and high FCR. Ultra‐brief FCR measures with a scale score, such as the recently validated single‐item screener FCR‐1,^38^ might be more appropriate. A future challenge is how to incorporate these FCR‐specific measures into routine oncology care, in order to ensure that cancer survivors are referred to adequate care.

### 4.2 Study limitations

The current study has several limitations. First, we evaluated the Dutch DT version, with several adaptations in the problem list compared to the original English version.^1^ While the DT score and fears item are identical, the results for the emotional domain score should be interpreted in relation to this specific version. Second, we included BCS and CRCS, and generalization to other cancer types should be made with caution. Third, the BCS sample had higher distress and FCR scores compared to the CRCS sample, and the percentage of BCS patients with high FCR was higher than found in a recent meta‐analysis.^39^ These high scores might reflect a selection bias as participants were willing to participate in a self‐management intervention study and were recruited 2–4 months after treatment. Explorative analyses in the two separate samples yielded similar conclusions. Fourth, the questionnaires in this study were used in a research context, which differs from the actual clinical screening context. Patients might answer differently when they know the questionnaires will be evaluated by their healthcare provider. Levels of distress might also be higher prior to or during a clinical appointment, while the assessments in these studies were not linked to a clinical appointment. Finally, there is no gold standard clinical interview for FCR that could be used as reference measure. Alternatively, there are multiple screening measures available^17^ amongst which the CWS‐6 and Fear of Cancer Recurrence Inventory severity subscale (FCRI‐SF) are often used. We evaluated the DT against the CWS‐6 which is a well‐established measure with two validated cut‐off scores.

## 5 CONCLUSION

Improving FCR detection becomes increasingly important to ensure that patients with elevated FCR have access to available evidence‐based interventions. This study evaluated the use of a widely implemented cancer‐specific distress screening tool, the DT and accompanying problem list, as a screening instrument for FCR. This study suggests that the DT is not suitable to detect FCR. Future research is needed to investigate how FCR‐specific measures can feasibly be integrated in routine oncology care.

## AUTHOR CONTRIBUTIONS

All authors were involved with the design of the study. Esther Deuning‐Smit performed the analyses and all authors contributed to the data‐interpretation. Esther Deuning‐Smit drafted the manuscript, which was reviewed and supplemented by the co‐authors Linda Kwakkenbos, José A. E. Custers, Rosella P. M. G. Hermens and Judith B. Prins. The final version of the manuscript was approved by all authors.

## CONFLICT OF INTEREST

The authors have no conflict of interests to declare.

## ETHICS STATEMENT

The present study used available data from two existing studies. These studies obtained ethical approval by the Radboud University Medical Center Medical Review Ethics Committee (No. 2009/144 and No. 2011/562).

## Supporting information

Supporting Information S1

## Data Availability

Data sharing is not applicable to this article as no new data were created or analyzed in this study.
