# Supplementary material for: Evaluating the capacity of the distress thermometer to detect high fear of cancer recurrence
Source: Psychooncology. 2022 Nov 24;32(2):266–74. doi: 10.1002/pon.6066 (PMC10099705; doi:10.1002/pon.6066)
Supplement: Supplementary file 1 — Supporting Information S1 [file PON-32-266-s001.docx]

***Supplementary table 1. Accuracy measures for a combination of any DT score and the fears item with the CWS-6 as reference standard***

| DT cut-off + fears item | CWS-6 **≥ 10** | | | | CWS-6 **≥ 12** | | | |
| --- | --- | --- | --- | --- | --- | --- | --- | --- |
|  | Sens | Spec | ppv | npv | Sens | Spec | ppv | npv |
| ≥1 | .274 | .955 | .902 | .462 | .423 | .945 | .805 | .753 |
| ≥2 | .274 | .966 | .925 | .464 | .423 | .952 | .825 | .754 |
| ≥3 | .259 | .966 | .921 | .459 | .397 | .952 | .816 | .746 |
| ≥4 | .230 | .977 | .939 | .453 | .346 | .959 | .818 | .732 |
| ≥5 | .193 | .977 | .929 | .441 | .295 | .966 | .821 | .718 |
| ≥6 | .126 | .989 | .994 | .424 | .192 | .979 | .833 | .693 |
| ≥7 | .074 | .989 | .909 | .410 | .115 | .986 | .818 | .675 |
| ≥8 | .030 | .989 | .800 | .399 | .051 | .993 | .800 | .661 |
| ≥9 | .007 | 1.000 | 1.000 | .396 | .013 | 1.000 | 1.000 | .653 |
| ≥10 | - | 1.000 | - | .395 | - | 1.000 | - | .650 |

***Supplementary table 2.* Accuracy measures for DT scores in the CRCS sample with the CWS-6 as reference standard**

|  | CWS-6 **≥ 10** | | | | CWS-6 **≥ 12** | | | |
| --- | --- | --- | --- | --- | --- | --- | --- | --- |
| DT cut-off | Sens | Spec | ppv | npv | Sens | Spec | ppv | npv |
| ≥1 | .929 | .227 | .433 | .833 | .875 | .179 | .23.3 | .833 |
| ≥2 | .893 | .409 | .490 | .857 | .813 | .321 | .255 | .857 |
| ≥3 | .786 | .591 | .550 | .813 | .813 | .518 | .325 | .906 |
| ≥4 | .536 | .727 | .556 | .711 | .563 | .679 | .333 | .844 |
| ≥5 | .464 | .795 | .591 | .700 | .500 | .750 | .364 | .840 |
| ≥6 | .393 | .886 | .688 | .696 | .438 | .839 | .438 | .839 |
| ≥7 | .250 | .909 | .636 | .656 | .313 | .893 | .455 | .820 |
| ≥8 | .179 | 1.000 | 1.000 | .657 | .250 | .982 | .800 | .821 |
| ≥9 | .071 | 1.000 | 1.000 | .629 | .125 | 1.00 | 1.00 | .800 |
| ≥10 | .071 | 1.000 | 1.000 | .629 | .125 | 1.00 | 1.00 | .800 |

***Supplementary table 3.* Accuracy measures for DT scores for the CWS-6 in the BCS sample with the CWS-6 as reference standard**

|  | CWS-6 **≥ 10** | | | | CWS-6 **≥ 12** | | | |
| --- | --- | --- | --- | --- | --- | --- | --- | --- |
| DT cut-off | Sens | Spec | ppv | npv | Sens | Spec | ppv | npv |
| ≥1 | .981 | .182 | .741 | .800 | .983 | .101 | .424 | .900 |
| ≥2 | .886 | .341 | .762 | .556 | .950 | .270 | .467 | .889 |
| ≥3 | .762 | .477 | .777 | .457 | .817 | .393 | .476 | .761 |
| ≥4 | .686 | .614 | .809 | .450 | .767 | .517 | .517 | .767 |
| ≥5 | .571 | .705 | .822 | .408 | .633 | .607 | .521 | .711 |
| ≥6 | .390 | .773 | .804 | .347 | .433 | .719 | .510 | .653 |
| ≥7 | .219 | .841 | .767 | .311 | .300 | .865 | .600 | .647 |
| ≥8 | .114 | .909 | .750 | .301 | .167 | .933 | .625 | .624 |
| ≥9 | .048 | .977 | .833 | .301 | .067 | .978 | .667 | .608 |
| ≥10 | .010 | 1.000 | 1.000 | .297 | .000 | .989 | .000 | .595 |

***Supplementary table 4.* Accuracy measures for emotional domain scores for the CWS-6 in the CRCS sample with the CWS-6 as reference standard**

| Emotional domain | CWS-6 **≥ 10** | | | | CWS-6 **≥ 12** | | | |
| --- | --- | --- | --- | --- | --- | --- | --- | --- |
|  | Sens | Spec | ppv | npv | Sens | Spec | ppv | npv |
| ≥1 | .897 | .535 | .565 | .885 | .889 | .444 | .348 | .923 |
| ≥2 | .759 | .791 | .710 | .829 | .778 | .685 | .452 | .902 |
| ≥3 | .690 | .837 | .741 | .800 | .722 | .741 | .481 | .889 |
| ≥4 | .414 | .907 | .750 | .696 | .611 | .907 | .688 | .875 |
| ≥5 | .310 | .977 | .900 | .677 | .500 | .981 | .900 | .855 |
| ≥6 | .172 | 1.000 | 1.000 | .642 | .278 | 1.000 | 1.000 | .806 |
| ≥7 | .138 | 1.000 | 1.000 | .632 | .222 | 1.000 | 1.000 | .794 |
| ≥8 | .069 | 1.000 | 1.000 | .614 | .111 | 1.000 | 1.000 | .771 |
| ≥9 | .034 | 1.000 | 1.000 | .606 | .056 | 1.000 | 1.000 | .761 |
| ≥10 | - | 1.000 | 1.000 | - | - | 1.000 | - | .750 |

***Supplementary table 5.* Accuracy measures for emotional domain scores for the CWS-6 in the BCS sample with the CWS-6 as reference standard**

| Emotional domain | CWS-6 **≥ 10** | | | | CWS-6 **≥ 12** | | | |
| --- | --- | --- | --- | --- | --- | --- | --- | --- |
|  | Sens | Spec | ppv | npv | Sens | Spec | ppv | npv |
| ≥1 | .886 | .318 | .756 | .538 | .933 | .247 | .455 | .846 |
| ≥2 | .771 | .500 | .786 | .478 | .867 | .427 | .505 | .826 |
| ≥3 | .686 | .682 | .837 | .423 | .783 | .562 | .547 | .794 |
| ≥4 | .505 | .795 | .855 | .402 | .650 | .742 | .629 | .759 |
| ≥5 | .400 | .909 | .913 | .388 | .517 | .831 | .674 | .718 |
| ≥6 | .276 | .909 | .879 | .345 | .383 | .888 | .697 | .681 |
| ≥7 | .190 | .955 | .909 | .331 | .283 | .944 | .773 | .661 |
| ≥8 | .143 | .977 | .938 | .323 | .233 | .978 | .875 | .654 |
| ≥9 | .076 | .977 | .889 | .307 | .133 | .989 | .889 | .629 |
| ≥10 | .038 | 1.000 | 1.000 | .303 | .067 | 1.000 | 1.000 | .614 |
